# Bipolar Ionization Did Not Reduce Airborne Bacteria in a Lecture Hall

**DOI:** 10.1021/acsestair.4c00235

**Published:** 2024-10-28

**Authors:** David A. Kormos, Nishit J. Shetty, Elliott T. Gall, Aaron J. Prussin, Amy Pruden, Linsey C. Marr

**Affiliations:** † Department of Civil and Environmental Engineering, Virginia Tech, Blacksburg, Virginia 24061, United States; ‡ Department of Civil, Environmental, and Architectural Engineering, University of Kansas, Lawrence, Kansas 66045, United States; § Department of Mechanical and Materials Engineering, Portland State University, Portland, Oregon 97201, United States

**Keywords:** Ionization, bioaerosol, ionizer, air cleaning, bacteria

## Abstract

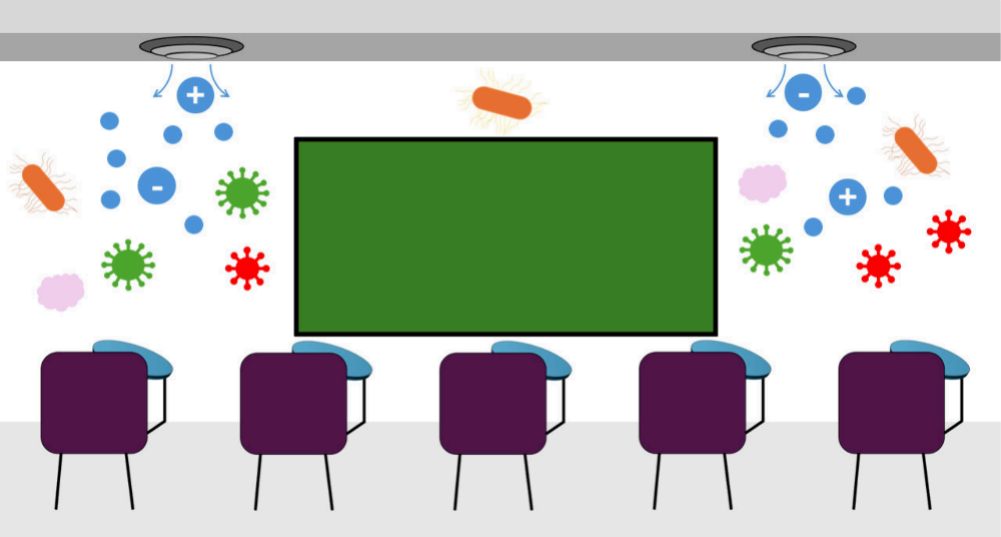

Ionization treatment of indoor air has attracted attention for its potential to inactivate airborne pathogens and reduce disease transmission, yet its real-world effectiveness remains unverified. We evaluated the impact of an in-duct, bipolar ionization system on airborne particles, including culturable bacteria, in a lecture hall. The ionizer was off with variable fan speed for 1 week, on with variable fan speed for a second week, and on with high and constant fan speed for a third week. We measured ion concentrations and aerosol particle concentrations, and we collected bioaerosol samples for analysis of 16S rRNA gene copies representing total bacteria and colony forming units (CFUs) on Tryptic Soy Agar representing culturable bacteria. There were no significant differences in positive, in-room ion concentrations between any weeks; however, negative, in-room ion concentrations were significantly lower when the ionizer was on with constant fan speed. To account for day-to-day variability in total bacteria concentrations, related to occupancy and other factors, we examined the ratio of CFUs to 16S rRNA gene copies (CFU gc^–1^) and found no significant differences whether the ionizer was on or off. This result indicates that the ionizer was not effective at reducing levels of culturable airborne bacteria in this study.

## Abstract 2

This study evaluates the effectiveness of an in-duct ionization system in a lecture hall, finding no significant difference in culturable airborne bacteria when the ionizer was on vs off.

## Introduction

One approach for reducing the risk of airborne transmission of disease in indoor spaces is to employ air-cleaning technologies that remove or inactivate pathogens.^1,2^ Ionization is an emerging air-cleaning technology that has attracted considerable attention because of its ease of installation and operation.^3−5^ Manufacturers’ claims and laboratory-based studies indicate its potential for enhancing removal of particulate matter and inactivating microorganisms in the air and on surfaces.^6−9^ Ionization has been implemented across diverse settings, including educational institutions, places of worship, and healthcare facilities.^3,10^ However, studies demonstrating its effectiveness as an air cleaning technology in real-world buildings occupied by humans are limited.

Bipolar ionization systems produce both positive and negative ions, mainly from water vapor, which then interact with other molecules and particles in the air. In theory, ions may attach to particles, enhance coagulation, and increase particle size, which could accelerate removal by gravitational settling and more efficient capture by air filtration systems.^3−5^ Microorganisms may be inactivated through the interaction of ions with membranes or surface proteins. For volatile organic compounds (VOCs), ions may enhance oxidation, producing secondary products.^11^ Due to this effect and other unintended consequences of ionization, its benefits and risks must be weighed carefully.

Ionization has been investigated in controlled studies in chambers and transportation vehicles.^3−5,12,13^ One study employed a chamber measuring 12 ft ×10 ft ×25 ft, equipped with a recirculating HVAC system, to evaluate the impact of bipolar ionization on the bacteriophage MS2 in the air and on surfaces.^3^ Total particle concentrations, size distributions, and deposition rates remained largely unchanged when comparing experimental and control conditions with the ionizer on and off, respectively. With the ionizer on and ion counts ranging from 1,000 # cm^–3^ to 6,000 # cm^–3^, an 87% reduction in MS2 concentration in air was observed at 60 min, while measurements at four other time points up to 120 min showed no difference. The study reports an equivalent clean air delivery rate of 58 m^3^ hr^–1^ (34 ft^3^ min^–1^) for the device. A study in a large, room-sized chamber reported net reductions of 34.4% to 100% for aerosolized influenza A and B viruses, human respiratory syncytial virus (RSV), and SARS-CoV-2 alpha and delta strains after 30 min.^12^ Another laboratory-based study demonstrated limited efficacy of ionization for removing ultrafine particulate matter (<0.15 μm) and an inconsequential effect on fine particulate matter (PM_2.5_) levels.^4^

A study conducted in an unoccupied tram in Spain revealed limited effectiveness of bipolar ionization.^13^ Concentrations of airborne bacterial colony forming units (CFUs) were reduced by 92% after 90 min when the tram’s filtration system was utilized, and the reduction marginally increased to 94% when both ionization and filtration were applied together. Similarly, in a comparative analysis in an unoccupied office setting, unipolar ionization modestly reduced particulate concentrations without ventilation, while bipolar ionization had minimal impact.^14^ Ventilation with filtration significantly lowered particle levels, with unipolar ionization providing a slight 6–10% enhancement, unlike bipolar ionization which showed no additional benefit. Boeing’s investigation into the technology found minimal or no discernible reductions in microbial presence on surfaces within laboratory and aircraft environments,^5^ in contrast to the device manufacturer’s claim of a reduction rate exceeding 95% for various pathogens. Such discrepancies can be at least partially explained by testing in small, sealed chambers and other shortcomings of laboratory studies that lead to overestimation of performance in real-world settings.^15^ Among the literature, some studies suggest that bipolar ionization can lead to a reduction in airborne particulate matter, attributed to enhanced particle deposition and filtration efficiency. Removal efficiency can vary based on many factors including filter material, particle concentration, flow rates, and humidity.^9,13,16−19^

While bipolar ionization devices have been studied in laboratory environments, the effectiveness of such devices in real-world settings remains largely unexplored. Here, we evaluated the effectiveness of an in-duct ionizer in a lecture hall during regular use. We tested the hypothesis that concentrations of culturable bacteria in air, normalized by total bacteria to account for day-to-day variability in loading, would be lower when a bipolar ionizer was ON versus OFF. In addition to collecting bioaerosol samples, we also measured ion concentrations and aerosol particle concentrations and size distributions to explore relationships among these variables. Results will contribute a more nuanced understanding of the practical effectiveness of bipolar ionization technology in managing airborne microorganisms, with implications for improving air quality in educational and other high-density indoor settings.

## Methods

**Figure 1:**
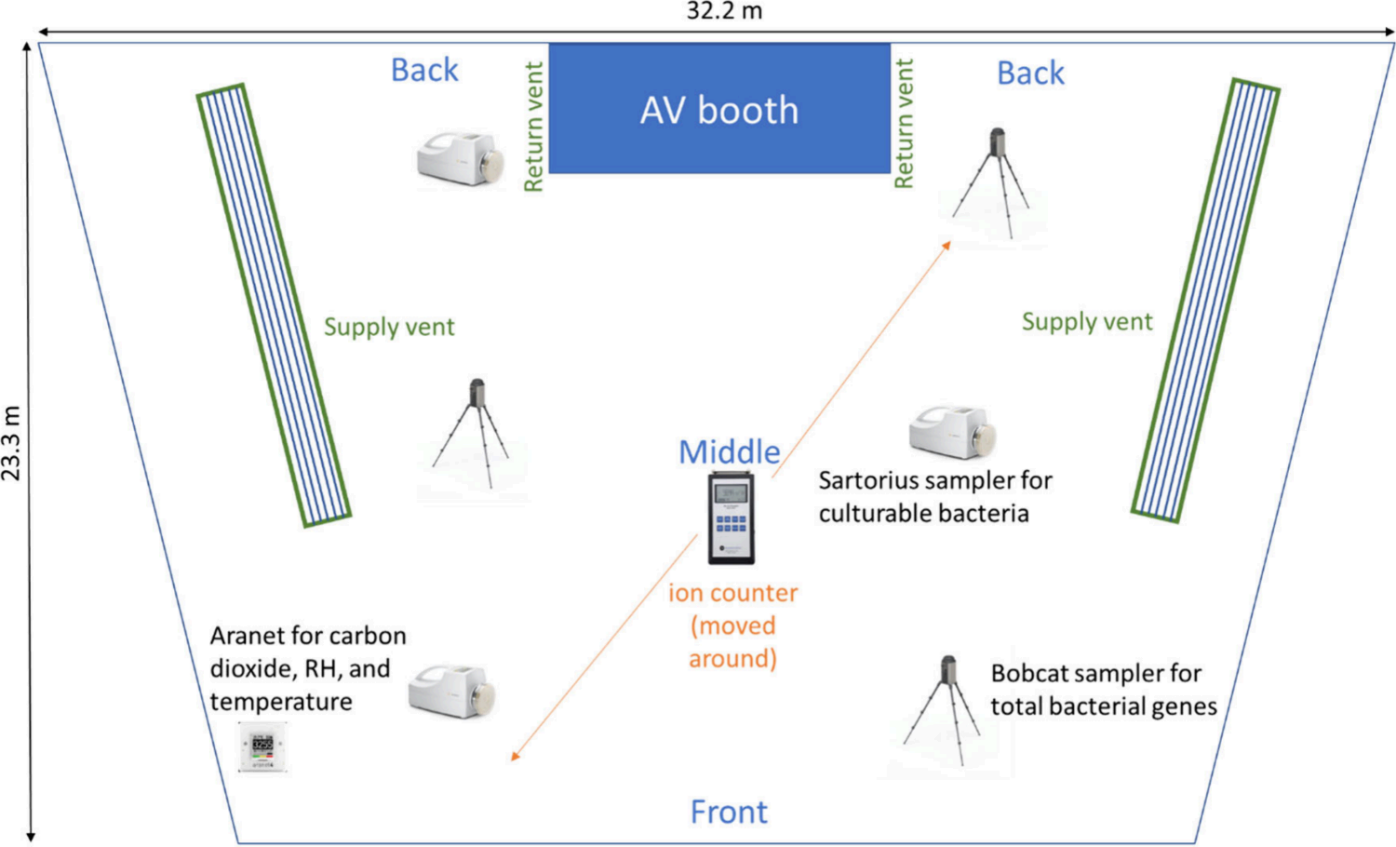
Layout of lecture hall showing sampling locations, ceiling supply vents, and return vents on the sides of the audiovisual (AV) booth. The ceiling height averages 7.3 m.

The study took place in a large lecture hall at Virginia Tech. The 540-seat hall is trapezoidal in shape, narrower at the front and wider at the back, as shown in Figure 1. Photographs of the room are available in Figure S1. The floor is carpeted, and the walls and ceiling panels are a mix of stained wood, painted surfaces, plastic, and fabric. Glass windows span the upper part of the back wall. The seats are constructed of metal, plastic, wood, foam, and fabric. Supply air vents are suspended from the ceiling on both sides of the hall, and return air vents are located on the sides of the audiovisual booth in the back of the hall. An HVAC system (Trane AHU-5) controls temperature in the lecture hall and adjoining lobby. During the study, the supply air flow rate varied between 104 and 272 m^3^ min^–1^, and the outdoor air fraction varied between 0 and 100%. The corresponding air change rate was 1.2–3.2 h^–1^. By default, the fan speed varied during the daytime to control temperature and was set to 35% of its maximum overnight. All supply air passed through MERV-13 filters. A bipolar ionization system was installed downstream of the filters in the HVAC system in October 2021. At the beginning of this study, the ionizer was inspected and cleaned.

Working in close collaboration with the university’s facilities team, we developed a sampling strategy to evaluate the impact of in-duct ionization system on bacterial load in the air. The study took place over 3 weeks in the fall of 2023, following a pilot study conducted in March 2023 to refine methods. For 1 week at a time, we operated the ionizer either OFF with variable fan speed during Monday 25 September through Friday 29 September, ON with variable fan speed during Monday 9 October through Friday 13 October, or ON with constant fan speed (90% of its maximum) during Monday 6 November through Friday 10 November. We conducted sampling in the lecture hall for 1 h each day. Due to the noise of the samplers, we could not use them during classes and instead scheduled sampling to occur during breaks in between classes, ideally immediately following periods of high occupancy. Sampling conditions and environmental parameters are summarized in Table 1.

**Table 1:** Conditions during Sampling over Three Five-Day (Mon-Fri) Periods^c^

Ionizer	Fan speed	Day	Occupancy^a^	In-duct CO_2_ (ppm)	In-duct RH (%)	In-room particles (# cm^–3^)	In-duct ions (# cm^–3^)	In-room ions, negative (# cm^–3^)	In-room ions, positive (# cm^–3^)
OFF	Variable	1	Medium	634 (95)	57.3 (1.0)	5152 (933)	50^b^ (NA)	2053 (133)	1850 (457)
		2	Medium	734 (111)	57.5 (1.5)	1767 (297)	50 (NA)	2479 (516)	2175 (679)
		3	Medium	678 (124)	57.7 (0.9)	2067 (405)	50 (NA)	2383 (154)	2290 (310)
		4	Low	514 (102)	57.8 (1.3)	9554 (596)	50 (NA)	2108 (372)	1936(98)
		5	High	682 (140)	57.0 (1.0)	5858 (1211)	50 (NA)	2901 (254)	3050 (629)
ON	Variable	6	Medium	573 (101)	46.5 (0.6)	2807 (459)	5939 (421)	2733 (616)	3117 (554)
		7	Medium	547 (79)	38.5 (0.3)	2376 (288)	2154 (481)	1950 (408)	2671 (311)
		8	Medium	675 (107)	42.1 (0.2)	2103 (173)	1457 (356)	1004 (489)	2061 (592)
		9	Medium	595 (92)	50.3 (0.6)	5937 (969)	9300 (3260)	1859 (492)	2334 (342)
		10	Medium	727 (116)	53.4 (0.6)	4634 (1752)	10580 (417)	2631 (133)	3705 (667)
ON	Constant	11	Low	614 (98)	32.3 (0.2)	9393 (271)	1910 (1065)	464(368)	1495 (377)
		12	Empty	481 (83)	50.2 (0.1)	17222 (751)	5884 (389)	1134 (317)	2515 (531)
		13	Medium	521 (92)	49.7 (0.4)	18733 (1046)	10805 (414)	1263 (182)	2577 (472)
		14	Empty	510 (93)	56.8 (1.2)	14413 (1061)	6687 (2893)	1311 (348)	3276 (691)
		15	High	656 (112)	48.6 (1.1)	2024 (224)	7612 (417)	2491 (211)	4735 (943)

a Occupancy inputs empty, low, medium, and high correspond to an empty room, 1–50 people, 51–100 people, and >100 people, respectively.

b During days 1–5, when the ionizer was OFF, in-duct ion readings were not available but were assumed to be 50 # cm^–3^ based on measurements from other periods when the ionizer was OFF.

c The standard deviation is shown in parentheses alongside the average.

We continuously monitored environmental conditions and HVAC parameters, including in-duct temperature, relative humidity (RH), fan speed, ion concentrations, and CO_2_ concentrations through data collected by the building automation system and in-room temperature, RH, and carbon dioxide (CO_2_) through a sensor (Aranet4) placed in the front of the lecture hall. In-duct ion concentrations were reported by a device from the same company that supplied the ionizer, and it is unclear whether they represent positive ions, negative ions, or the sum of both. Because the CO_2_ and RH data sets from the Aranet4 were incomplete (a few days were missing), we relied on in-duct CO_2_ and RH data in the analysis. The linear correlation coefficients between the two for CO_2_ and RH were very strong (*r* = 0.91 and *r* = 0.93 respectively). During each 1-h sampling period, we measured concentrations of particles in the size range of 0.3–25 μm (TSI AeroTrak 9303) at 1 min frequency in the middle of the room and ion concentrations (Air Ion Counter Model AIC2), alternating between positive and negative ions every 5 min at different locations in the room. We grounded the ion counter to a power outlet with a ground terminal during all measurements in the lecture hall. We estimated occupancy in four categories of empty, low, medium, and high corresponding to an empty room, 1–50 people, 51–100 people, and >100 people, respectively. The room was never close to full occupancy of 540. Samples were collected at the same time on Mondays, the same time on Tuesdays, etc., but these times differed by day of week. Room occupancy prior to sample collection varied, even at the same time and day of week, due to factors such as examinations, sessions held remotely, and occasional low attendance. Outside the sampling period (i.e., during the other 23 h of the day), the ion counter was placed at the front of the room and programmed to monitor negative ions exclusively.

To examine spatial variability, we collected bioaerosol samples at the back, middle, and front of the lecture hall, as shown in Figure 1. We used three portable high-volume samplers (ACD-200 Bobcat) at a flow rate of 200 L min^–1^ for 1 h to collect 12 m^3^ of aerosol for analysis of total bacterial gene copies by quantitative polymerase chain reaction (qPCR). After collection, we immediately eluted the samples into ∼7 mL of phosphate buffered saline (PBS) using the manufacturer’s kit. We used three hand-held samplers (Sartorius MD8 Airport) at a flow rate of 50 L min^–1^ for 30 min to collect 1.5 m^3^ of aerosol onto gelatin filters for culture-based analysis of microbes. Figure 1 shows the locations of these samplers in the lecture hall.

Immediately following the sampling period, we transported the eluted samples to the laboratory on ice and stored them at −80 °C. We filtered eluents from the Bobcat samplers through 0.22 μm pore filters and then used the FastDNA Spin Kit for Soil to extract DNA directly from the filters. We performed qPCR in triplicate on all DNA extracts using the CFX96 Touch Real-Time PCR Detection System (BioRad Laboratories, Hercules, CA) to quantify initial concentrations of total bacterial 16S rRNA genes using SsoFast Evagreen Supermix (BioRad Laboratories, Hercules, CA) and universal primers.^20^ To ensure consistency and reliability, we processed samples from the same day of week and different ionizer operational status together on the same qPCR plate. Additional details about the qPCR protocol are provided in the Supporting Information (SI).

We transferred gelatin filters onto Tryptic Soy Agar (TSA) plates under aseptic conditions and incubated them at 37 °C for 24 h for assessment of bacterial culturability. TSA was selected for its ability to cultivate a wide range of heterotrophic microbes. Following incubation, we manually counted colonies, took photos of the plates (Figure S2), and then stored them. For plates with overgrown colonies (likely due to fungi), we counted half the area of the plate and then doubled the count, assuming symmetry in the plating. To calculate airborne concentrations of culturable bacteria, we divided the counts by the volume of air sampled (1.5 m^3^), generating results in terms of colony forming units per cubic meter (CFU m^–3^).

Using R, we employed the Kruskal–Wallis test and used analysis of variance (ANOVA) to determine differences in bacterial load and ion and particle concentrations respectively across the various operational states of the ionizer and locations of the samplers. Upon finding significant effects, we employed post hoc tests to pinpoint the specific conditions under which the bacterial load varied significantly. The Kruskal–Wallis test was conducted with a 95% confidence interval, and the Dunn’s post hoc tests were adjusted for multiple comparisons using the Bonferroni correction to control for Type I error. Similarly, the ANOVA was conducted with a 95% confidence interval, and post hoc comparisons were adjusted for multiple testing using the Tukey method to control for Type I error. We calculated Spearman correlation coefficients to identify associations between environmental conditions and bacterial presence in the air. We defined a threshold for significance at a p-value of 0.05.

## Results

To determine the effectiveness of an ionizer in a lecture hall, we measured concentrations of ions and bacteria in the air when the ionizer was OFF (days 1–5), ON with variable fan speed (days 6–10), and ON with constant fan speed (days 11–15). On day 11, we discovered that the ionizer was not functioning properly, and the facilities engineering team reset it in the middle of day 12. This malfunction was restricted to the beginning of the third week and did not affect the second week of sampling. We collected measurements and samples at the back, middle, and front of the lecture hall to determine whether differences in residence time—greater at the back near the return vents—were reflected in the observations.

**Figure 2:**
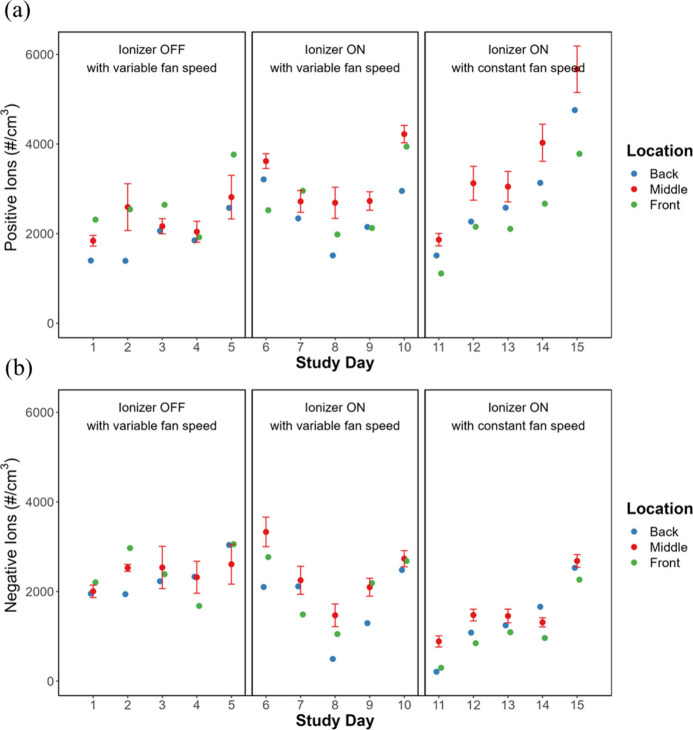
In-room positive (a) and negative (b) ion concentrations during the sampling period. Error bars represent one standard deviation and are shown for the middle location only to maintain legibility. Error bars are similar in magnitude for the other locations.

Figure 2 shows the in-room ion concentrations during the sampling hour for both positive and negative ions. Positive in-room ion concentrations ranged from 1,500 to 5,000 # cm^–3^ with no significant spatial variation between back, middle, and front of the room, and no significant difference by ionizer and fan status. Negative in-room ion concentrations were slightly lower, typically ranging from 1,000 to 3,000 # cm^–3^, with no significant spatial variation for the weekly data. Surprisingly, the negative in-room ion concentrations were significantly lower when the ionizer was ON with constant fan speed compared to the other two conditions. In-duct ion concentrations varied between 40 and 11,000 # cm^–3^, the maximum range of the sensor, and were significantly higher during the 2 weeks the ionizer was ON than when it was OFF (Table 1). Table S1 shows p-values for differences in ion, particle, and bacteria concentrations by ionizer status and location in the lecture hall, while Table S2 shows the posthoc results.

Ionization has the potential to enhance coagulation of particles, causing them to increase in size and deposit more rapidly, leading to lower concentrations, but we did not observe such an effect. Figure S3 shows the particle size distribution in five size bins ranging from 0.3 and 10 μm. The geometric mean diameter of airborne particles remained relatively stable between 0.40 and 0.42 μm, regardless of ionizer and fan status. Surprisingly, total particle concentrations were higher when the ionizer was ON with constant fan speed compared to the other two conditions. When the ionizer was OFF, the average concentration of particles >0.3 μm was 4880 # L^–1^, and when it was ON with constant fan speed, the average was 3572 # L^–1^. In contrast, concentrations were highest when the ionizer was ON with constant fan speed, an average of 12,357 # L^–1^ and a maximum of 18,000 # L^–1^. This observation contradicts the purported impact of ionization, but many other factors discussed below can affect particle concentrations.

**Figure 3:**
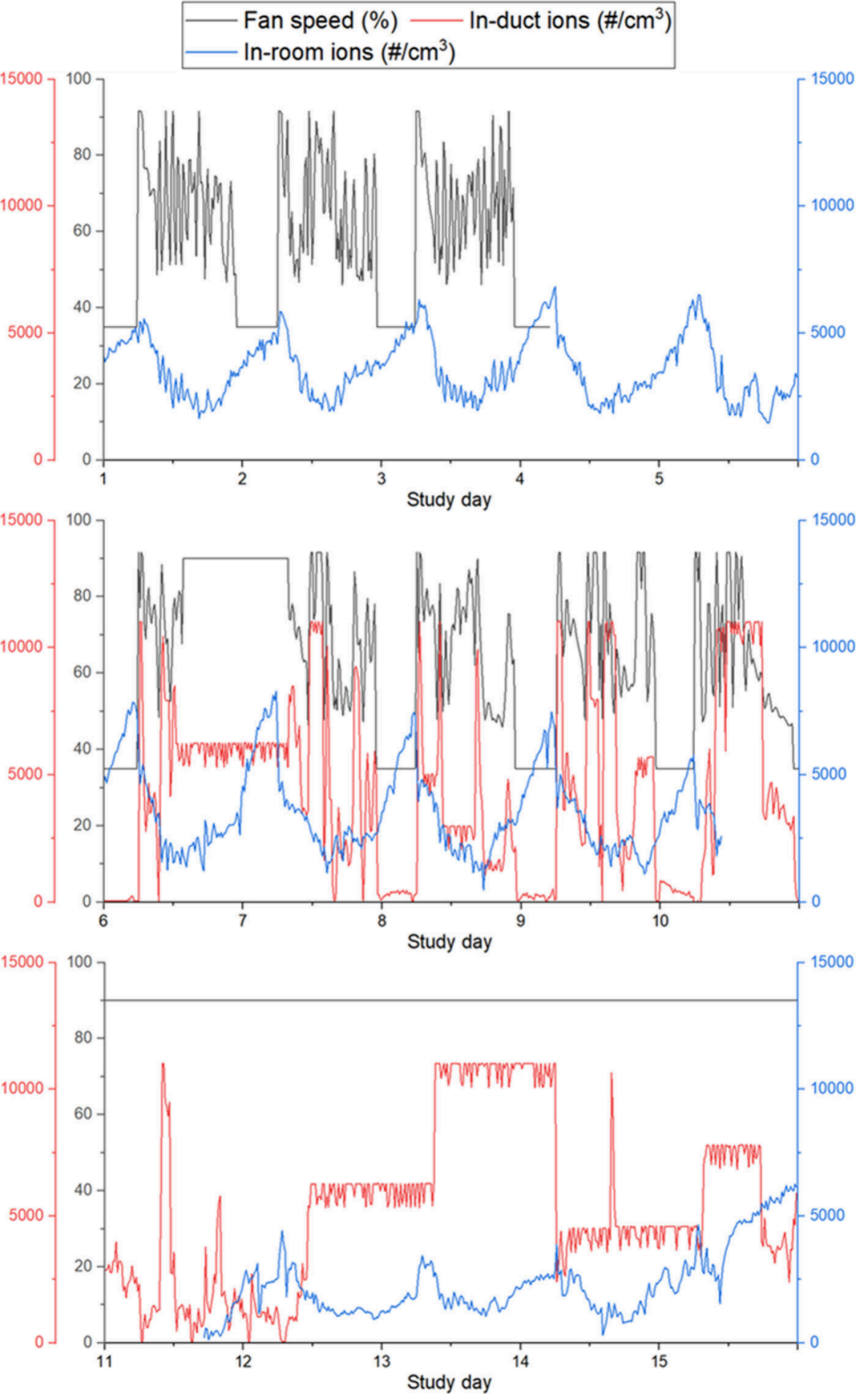
Time series of ion concentrations and fan speed in the air handling unit. The in-room concentration shoes negative ions. During days 1–5, when the ionizer was OFF, in-duct ion readings were not available but were assumed to be 50 # cm^–3^ based on measurements from other periods when the ionizer was OFF.

Figure 3 shows time series of fan speed, in-duct negative ion concentrations, and in-room negative ion concentrations over 24 h of each day of the study. In-duct ion concentrations were not available on study days 1–5 when the ionizer was OFF and were presumed to be 50 # cm^–3^, as observed during the pilot study under the same conditions. In-room ion concentrations appeared to be affected more by fan speed than by ionizer status. They exhibited a pronounced diurnal pattern, with levels escalating overnight to roughly 7000–9000 # cm^–3^, regardless of the ionizer’s status during the first 2 weeks when the fan speed was variable during the daytime and constant at 35% overnight. When the ionizer was ON with variable fan speed, in-duct ion concentrations appeared to be correlated with fan speed. The nighttime increase in ion concentrations is more apparent in Figure S4, which shows negative ion concentrations over a typical 24-h period when the ionizer was OFF. During the third week, the fan speed was held at 90%, and in-room ion concentrations were mostly lower than during the other 2 weeks, even though the ionizer was ON. After the ionizer was reset on day 12, in-duct ion concentrations were higher and remained approximately constant for 6–12 h at a time, with several step changes occurring for unknown reasons. In-room and in-duct ion concentrations were weakly negatively correlated (*r* = −0.32, *p* < 0.05).

**Figure 4:**
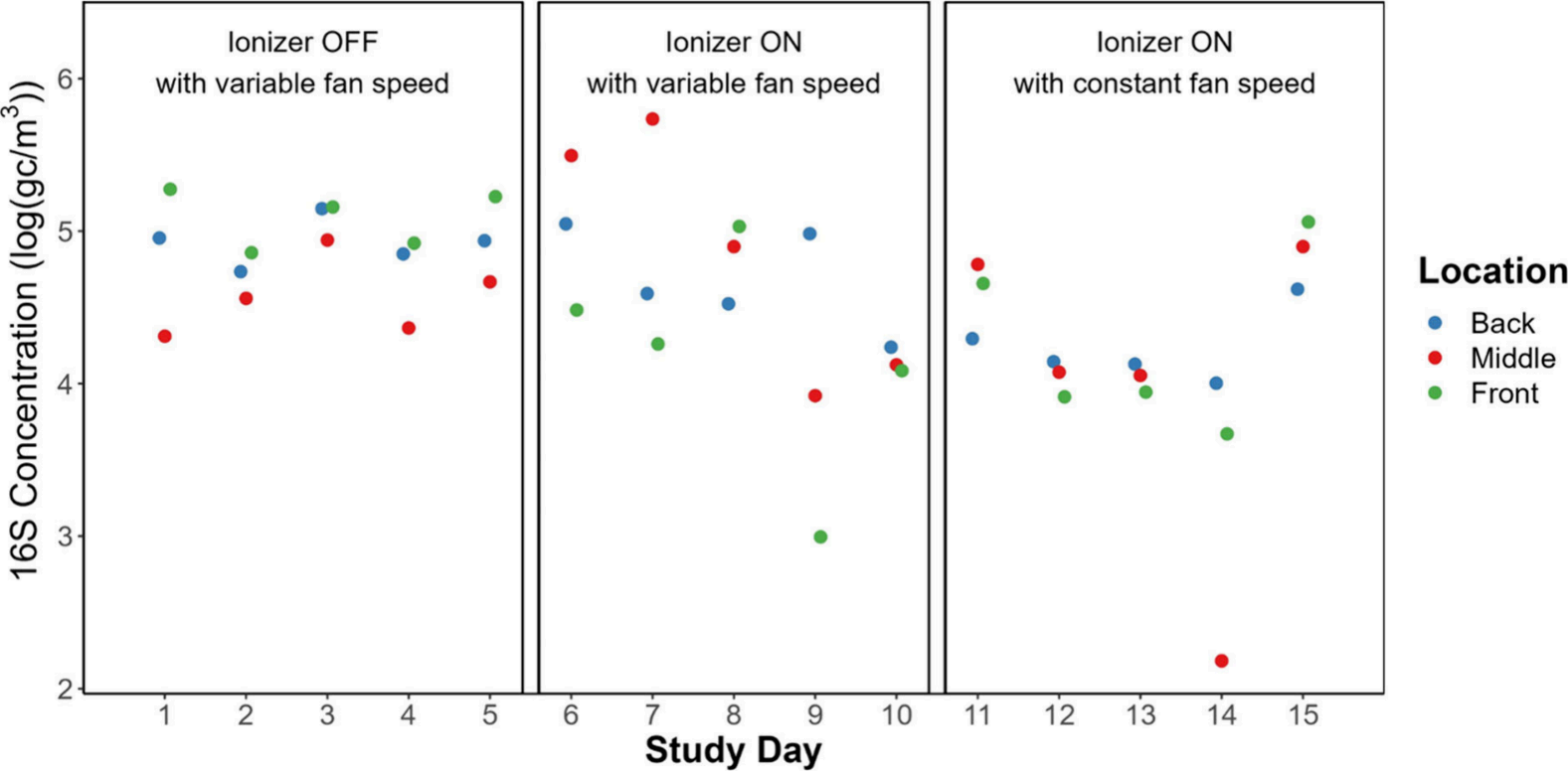
Concentrations of 16S rRNA gene copies in air samples collected for 1 h each day. Figure S5 shows results plotted on a linear scale.

On most days, concentrations of total bacteria in terms of the 16S rRNA gene ranged from 5 × 10^4^ to 2 × 10^5^ gene copies per cubic meter of air (gc m^–3^), as shown in Figure 4. Figure S5 shows 16S rRNA gene concentrations on a linear scale. 16S rRNA gene concentrations were not significantly different by location in the lecture hall (Table S1), although they were notably higher in the middle of the room, where most occupants sat, on days 6 and 7. When the ionizer was ON with constant fan speed during days 11–15, concentrations were lower, reaching a minimum of 1 × 10^2^ gc m^–3^. This decrease coincided with reduced room occupancy, notably on days 12 and 14, when occupancy was zero prior to sampling. 16S rRNA gene concentrations were significantly lower when the ionizer was ON with constant fan speed than when it was OFF (Table S2). The difference could be due to factors other than ionizer status, including a higher filtration rate resulting from a high and constant fan speed, as discussed below.

**Figure 5:**
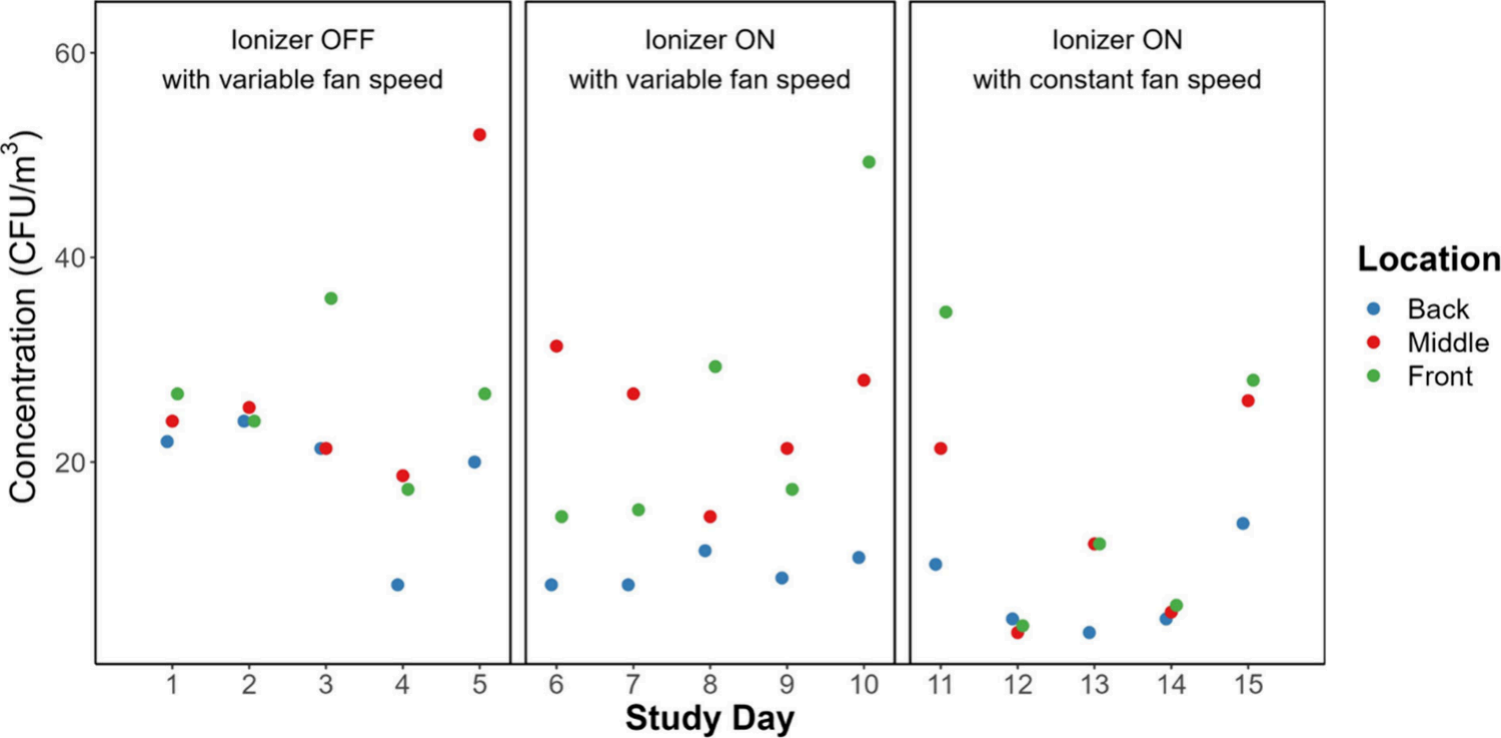
Concentrations of culturable bacteria on Tryptic Soy Agar plates in terms of colony forming units (CFU) in air samples collected for 1 h each day.

The range of concentrations of culturable bacteria in air, depicted in Figure 5, remained relatively consistent from week to week, registering between 30 to 40 CFU m^–3^ on most days. However, on days with zero occupancy, the concentrations were below 10 CFU m^–3^. Ionizer status and spatial variation both appeared to significantly impact CFU concentrations. The posthoc test identified significant spatial variation between the front and back, and the middle and back areas. A detailed examination revealed that during the week the ionizer was ON with variable fan speed, the concentration of culturable bacteria in the back of the room was significantly lower than in the middle and front of the room. Additionally, the concentration was significantly lower when the ionizer was ON with constant fan speed than when it was OFF, correlating with decreased occupancy within the room as well.

**Figure 6:**
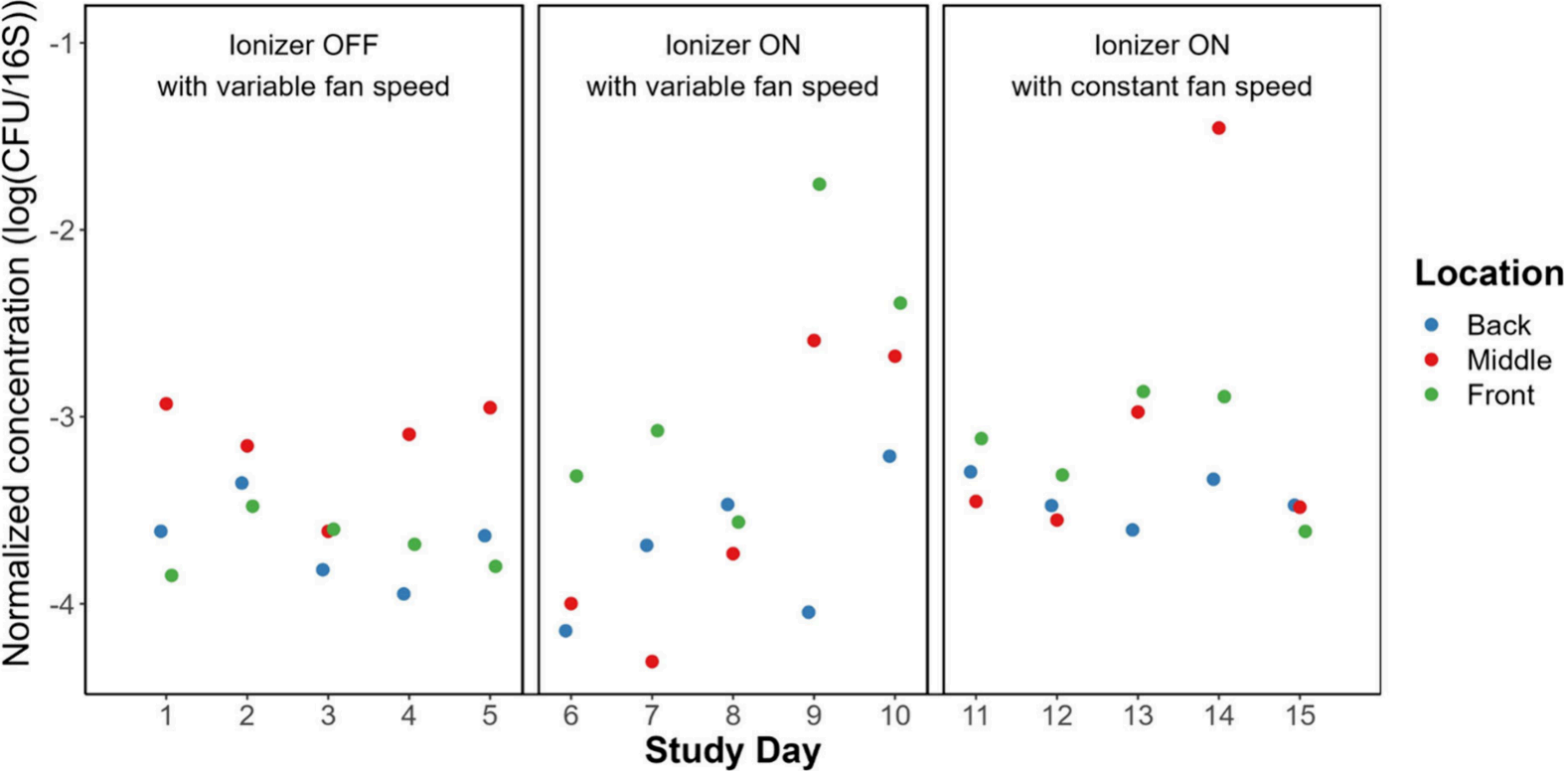
Concentrations of culturable bacteria on Tryptic Soy Agar plates in terms of colony forming units (CFU) normalized to 16S rRNA gene concentrations in air samples collected for 1 h each day. Figure S6 shows these results plotted on a linear scale.

To account for day-to-day variations in microbial biomass due to variable occupancy and other factors, we normalized concentrations of CFU to those of 16S rRNA gene copies (i.e., CFU gc^–1^). Figure 6 shows these ratios on a logarithmic scale, while Figure S6 shows them on a linear scale. Overall, the ratio was not significantly different across ionizer operational status. Day 14 exhibited a value nearly 2 orders of magnitude higher, which is likely attributable to zero occupancy in the room, leading to very low CFU and 16S rRNA values, with the latter nearing the limit of quantification. Conversely, days 9 and 10 stood out with values approximately 1 order of magnitude higher than the general trend, despite medium occupancy levels. Even after normalization, these days showed elevated CFU gc^–1^ ratios compared to other days. These values were not significantly different by location within the room.

**Figure 7:**
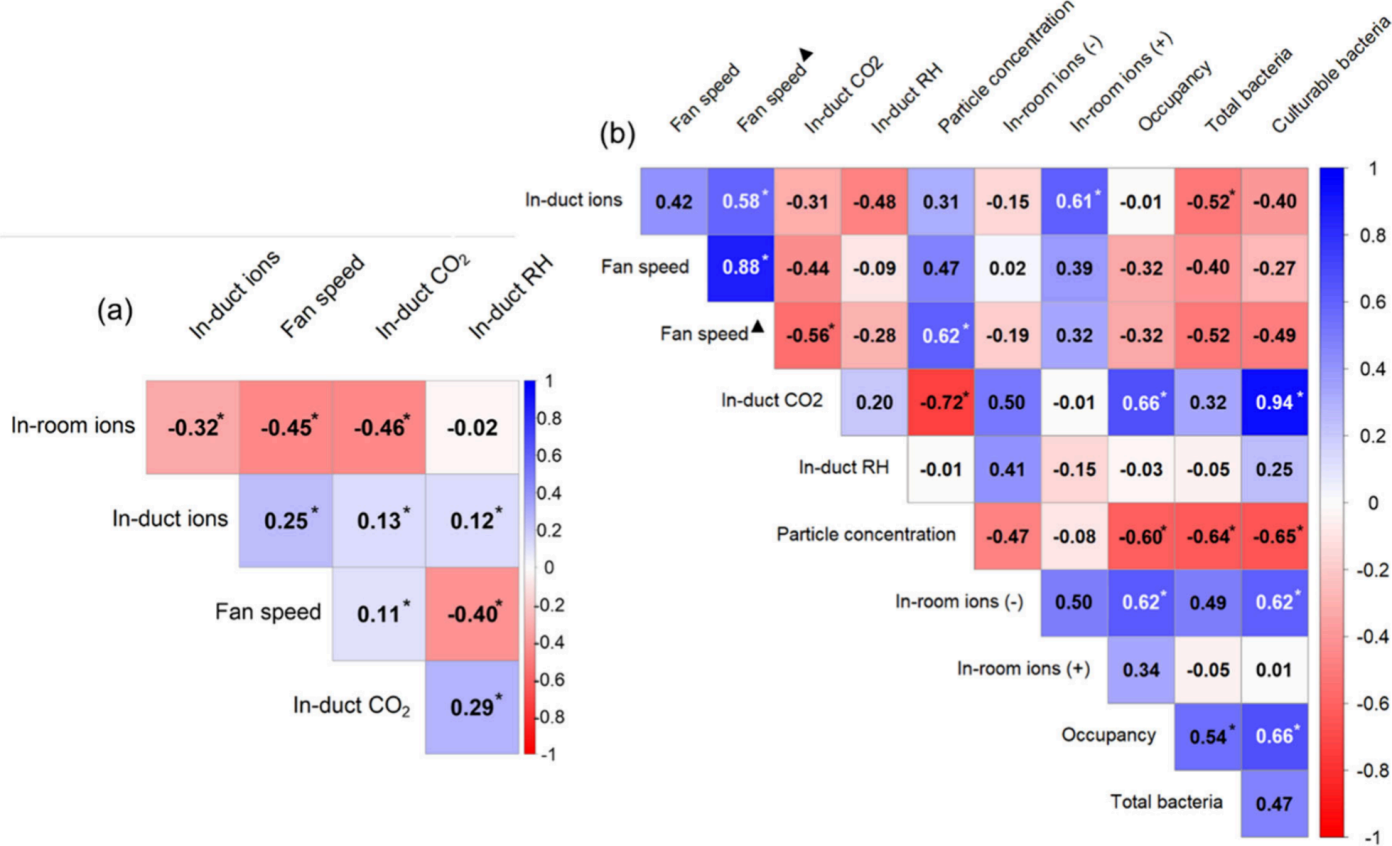
Correlation plots for variables measured (a) continuously 24 h per day and (b) only during the 1-h sampling period. Total bacteria are quantified in terms of 16S rRNA gene copies. Asterisk (*) denotes significant correlations (p*<*0.05). Triangle (▲) denotes measurements from the hour before sampling. In-duct ion measurements may represent positive or negative ions or both.

Figure 7 presents two correlograms that provide insights into the relationships between environmental variables over two different time frames: continuously over 24 h during each day of sampling (Figure 7a), and the 1-h periods of bioaerosol sampling (Figure 7b). Additionally, in Figure 7b, we included the fan speed during the 1 h prior to sampling, as the room was occupied during that time. This consideration is important because the fan speed while the room was occupied may impact bioaerosol concentrations. Figure S7 shows p-values for the correlations. There does not appear to be a strong correlation between RH and any factors, though there is a slight negative correlation with fan speed and a positive correlation with in-room CO_2_, both of which are significant.

Measurements during the hour of bioaerosol sampling revealed several notable correlations (Figure 7b). A strong positive correlation was observed between in-duct CO_2_ and culturable bacteria. Conversely, CO_2_ exhibited a strong negative correlation with particle concentration. Culturable bacteria were moderately negatively correlated with particle concentration, and moderately positively correlated with negative in-room ions, fan speed prior to the sampling period, and occupancy. Total bacteria were moderately negatively correlated with in-duct ion concentrations and particle concentration, and moderately positively correlated with fan speed prior to the sampling period and occupancy. Interestingly, while negative in-room ions showed no significant correlation with in-duct ions, positive in-room ions demonstrated a significant positive correlation with negative in-duct ions. This highlights a differential behavior in ion concentration dynamics within the room versus the duct environment.

## Discussion

In our real-world study of ionization in a lecture hall, ionization did not have a significant effect on the amount of culturable bacteria in the air. Although ionization has been shown to enhance removal of particles and inactivate microorganisms in some laboratory studies,^3−5,12,13^ we did not observe such an effect in this real-world setting. The Spanish tram study reported a similar finding, where the addition of ionization reduced culturable bacteria concentrations by only two additional percentage points compared to filtration alone.^13^ Similarly, Boeing’s investigation into the technology found minimal or no discernible reductions in microbial presence on surfaces within laboratory and aircraft environments.^5^ Another study demonstrated limited efficacy of ionization in removing ultrafine particulate matter and an inconsequential effect on PM_2.5_ levels.^4^

We estimated the effectiveness of the ionization system as an intervention in terms of inactivation efficiency: (1 – *C*_*ON*_/*C*_*OFF*_) × 100%, where *C*_*ON*_ and *C*_*OFF*_ are the normalized CFU-to-gene-copy concentrations when the ionizer was ON and OFF, respectively. A value of 100% means that the ionizer led to complete inactivation of culturable bacteria, and a value of 0% means that the ionizer led to no change in culturable bacteria. A negative value means that the normalized amount of culturable bacteria was higher when the ionizer was on. Averaging normalized CFU-to-gene-copy concentrations across days and locations for each week to obtain values of *C*_*on*_ and *C*_*off*_, we found inactivation efficiencies of −370% or −580% when the ionizer was ON with variable fan speed or ON with constant fan speed, respectively. In other words, the normalized bacteria concentrations were 3.7 and 5.8 times higher when the ionizer was ON compared to OFF. We interpret our finding of a negative efficiency of this intervention as an indicator that other factors besides ionization drive airborne bacteria concentrations, rather than a sign that the ionizer actually leads to an increase in culturable bacteria in the air.

Multiple factors affect concentrations of airborne bacteria indoors, such that any effect of ionization was not apparent in this study. As people are the major source of bioaerosols indoors, we expect lower concentrations of total bacteria when occupancy is lower. Among the factors we measured, the most pronounced and significant correlation was between in-duct CO_2_ and culturable bacteria (r = 0.94), indicating a strong association between people and microbial presence as quantified by CFUs. Room occupancy also showed a moderate and significant correlation with both total (r = 0.54) and culturable (r = 0.66) bacteria concentrations, reinforcing the premise that the number of people present is a key factor influencing bacterial load in indoor air. Concentrations of both total and culturable bacteria were lower when the ionizer was ON with constant fan speed compared to OFF with variable fan speed. This may be due to a combination of greater removal by filtration, thanks to a higher flow rate through the HVAC system’s filters, and lower occupancy. On days with equivalent occupancy levels during the week where the ionizer was ON with constant fan speed (specifically study days 11 and 15), the concentrations of total and culturable bacteria remained consistent with those observed during the other weeks, as depicted in Figures 4 and 5. Additionally, to account for day-to-day differences in total bacterial load, we calculated the ratio of culturable bacteria to total bacteria. The ratio was not significantly different by ionizer operational status, as depicted in Figure 6, indicating that ionizer status had no impact on the bacterial load in the room.

Interestingly, concentrations of in-duct ions and negative in-room ions were not significantly correlated during the 1-h sampling period. Upon entering the lecture hall from the supply duct, ions would have rapidly interacted with gas molecules, particles, and fixed surfaces by the time they reached the middle of the room. However, during the 1-h sampling period, in-duct negative ions and in-room positive ions were moderately correlated (r = 0.61). In-room negative ions demonstrated a slight positive correlation with culturable (r = 0.66) bacteria, while in-room positive ions were not correlated with any biological metrics. In-duct ions exhibited a slight negative correlation with total bacteria (r = −0.52). This pattern may be linked to the study days with no occupancy when the ionizer was ON with constant fan speed. The variability of in-duct ion concentrations also appeared to be affected by fan speed, as shown in Figure 3. With variable fan speed, the in-duct ions displayed greater fluctuation, whereas during the last week of sampling, other than the days noted where the ionizer seemed to not be functioning correctly, the in-duct ion concentration remained relatively stable for multiple hours at a time.

Ions in air may be lost through recombination of oppositely charged ions, attachment to aerosol particles and other surfaces, and chemical reactions.^21^ We estimated the first-order ion loss coefficient in the lecture hall by performing an ion balance on days 12 and 13, during the period when in-duct ion concentrations were ∼6000 # cm^–3^ and in-room ion concentrations were ∼2000 # cm^–3^ (Figure 3). Assuming well-mixed steady-state conditions and an air change rate of 3.2 h^–1^, we calculated a loss coefficient of 0.0018 s^–1^, corresponding to a characteristic time of 9 min. For comparison, the ion–ion recombination rate coefficient in the troposphere has been reported to be 1.0–3.0 × 10^–12^ m^3^ s^–1^,^22^ corresponding to a first-order rate coefficient of 0.002–0.006 s^–1^ at an ion concentration of 2000 # cm^–3^. The similarity in magnitude of this coefficient and the one estimated for ion loss in the lecture hall suggests that ion–ion recombination, occurring on the time scale of minutes, could be one fate of ions entering the room.

The prominent pattern of higher in-room ion concentrations overnight even when the ionizer was OFF (Figure 2) indicated another source of ions. The lecture hall is located below ground level in a geographic region with high levels of naturally occurring radon, a radioactive gas that generates ions as it decays. To ascertain the influence of environmental and room conditions on ion concentrations, we measured ion concentrations in other rooms and buildings, including a basement, a fourth-floor classroom, a ground-level floor, and an outdoor setting. As shown in Figure S8, ion concentrations were highest in the lecture hall and in the basement room in a different building, thus supporting our hypothesis that radon contributed to elevated ion levels. Additionally, observations from our preliminary and pilot tests indicated that prolonged operation could lead to the accumulation of dust on the ionizer, diminishing its efficacy.

This study has several limitations. The findings are specific to a single, large lecture hall and may not be generalizable to other settings with different designs, occupancy patterns, and HVAC systems. Our particle characterization method was limited to a single, portable optical particle counter, and we did not examine the potential formation of secondary particles through reactions of ions with organic compounds. Our data on outdoor air (OA) fraction were incomplete. Such data, combined with indoor and outdoor ion and particulate matter concentrations, could have provided valuable insights into the observed variations in ion concentrations. The sampling strategy, constrained to breaks between classes, might not fully capture bioaerosol variability during high occupancy periods. The three-week duration of the study may not capture long-term trends or seasonal variations, and weekly changes in ionizer operation might not account for cumulative effects. We also acknowledge that culture media are limited in the range of viable microbes that they are able to support. Lastly, the statistical methods used have inherent limitations that could affect the validity of the conclusions.

Our study evaluated the impact of an in-duct, bipolar ionization system on airborne bacteria but not viruses. Sampling and enumerating airborne viruses present greater challenges compared to bacteria due to their smaller size, requirement of specific host cells for culturing, and lack of a common gene.^23^ Bacteria, being larger and having more complex cell structures, are thought to be more susceptible to physical and chemical inactivation methods. Given that the ionization system was not effective at reducing culturable airborne bacteria, it is plausible that it may be even less effective against viruses. Despite these differences, some aspects of our findings may still be applicable to viruses. For instance, the ionization process generates reactive oxygen species (ROS) that can damage microbial cell walls and viral envelopes,^24,25^ although the efficiency of this mechanism can vary widely between different types of pathogens. One study conducted in a chamber found that viruses were inactivated at a higher rate than bacteria under certain conditions.^12^ Therefore, while our results indicate limited effectiveness against bacteria, further research is needed to conclusively determine the impact on airborne viruses. Future studies should aim to directly measure the impact of ionization systems on airborne viruses in real-world settings, for example by aerosolizing a nonpathogenic virus into a room and evaluating its fate. This would provide a more comprehensive understanding of ionization’s effectiveness across different types of airborne pathogens.

Future studies of ionization systems should consider incorporating measurements of OA fractions and outdoor PM concentrations. This could be achieved by utilizing data from nearby air quality monitoring networks, such as PurpleAir or regulatory monitors, to estimate the contribution of outdoor particles to indoor environments. Additionally, understanding the relationship between OA fractions, ambient particle levels, and ion concentrations, both indoor and outdoor, could help shed light on the mechanisms driving the observed ion behavior and improve the effectiveness of ionization treatments in real-world settings. Further research in diverse settings and over longer periods is needed to validate and extend these findings.

Our study of the effectiveness of ionization in a lecture hall reveals that the air-cleaning technology did not achieve the goal of reducing airborne concentrations of bacteria under real-world conditions. Rather, factors such as occupancy and HVAC operation emerged as the primary drivers of bacterial load in the air. Our results underscore the complexity of the indoor environment and show that demonstration of the efficacy of air-cleaning technologies in the laboratory does not necessarily translate to real-world settings. Given the lack of evidence that ionization is effective in real-world settings and concerns about the unintended consequences of chemical reactions initiated by ions, we caution against the use of ionization unless further studies produce different findings.

## Supporting Information Available

The 16s rRNA qPCR protocol; the p-values for different variables of interest by ionizer status and location and the p-values for associated posthoc tests; photographs of the lecture hall; examples of the bacterial growth seen on plates; particle number concentrations by size; example negative ion concentrations for a 24 h period; 16s rRNA and normalized bacteria concentrations plotted on linear scale; p-values for correlation plots; negative ion concentrations in other rooms and buildings on campus (PDF)
