# Supplementary material for: Bipolar Ionization Did Not Reduce Airborne Bacteria in a Lecture Hall
Source: ACS EST Air. 2024 Oct 28;1(12):1696–705. doi: 10.1021/acsestair.4c00235 (PMC11650565; doi:10.1021/acsestair.4c00235)
Supplement: Supplementary file 1 — ea4c00235_si_001.pdf [file ea4c00235_si_001.pdf]

Bipolar Ionization Did Not Reduce Airborne Bacteria in a Lecture Hall

David A. Kormos[1], Nishit J. Shetty[1,2], Elliott T. Gall[3], Aaron J. Prussin II[1], Amy Pruden[1], Linsey C. Marr[1*]

[1] Department of Civil and Environmental Engineering, Virginia Tech, Blacksburg, Virginia 24061

[2] Department of Civil, Environmental, and Architectural Engineering, University of Kansas, Lawrence, Kansas 66045

[3] Department of Mechanical and Materials Engineering, Portland State University, Portland, Oregon 97201

[*]Corresponding author; lmarr@vt.edu

16s rRNA qPCR protocol:
The 10 µl reaction mixture consisted of 2x SsoFast EVAgreen Supermix (BioRad Laboratories, Hercules, CA), 1 µl of DNA template, 400 nM primers, and molecular grade water. Triplicate technical replicates of each sample were amplified along with triplicate standard curves and a negative control (molecular grade water). Samples were only accepted if they had a standard deviation under 15% between technical replicates. qPCR was carried out on a CFX Connect TM Touch Real-Time PCR Detection System (BioRad Laboratories Hercules, CA). The protocol consisted of 1 cycle of 98°C for 2 min, 40 cycles of 98°C for 5 s and annealed 55°C for 5 s before a final melt curve stage with temperature ramping from 55℃ to 95℃ with 0.5℃ per read.

Table S1. p-values for variables of interest by ionizer status and location. $p<0.05$ shown in red.

Variable	Ionizer status	Location	Test
Negative ions	0.00	0.48	ANOVA
Positive ions	0.12	0.16	ANOVA
Particles	0.02	N/A	ANOVA
16S rRNA genes	0.00	0.88	Kruskal-Wallis
CFU	0.01	0.01	Kruskal-Wallis
CFU/16s rRNA	0.17	0.08	Kruskal-Wallis

Table S2. p-values of post-hoc tests differences between variables of interest. p<0.05 shown in red.

Variable	Back - front	Back - middle	Middle - front	OFF – ON variable	OFF - ON constant	ON - ON constant	Test
Negative Ions	0.95	0.48	0.65	0.31	0.00	0.01	Tukey
Positive Ions	0.84	0.15	0.38	0.27	0.12	0.90	Tukey
Particles	N/A	N/A	N/A	0.89	0.05	0.02	Tukey
16S rRNA	1.00	1.00	1.00	0.33	0.00	0.23	Dunn
CFU	0.01	0.03	1.00	0.71	0.01	0.20	Dunn
CFU/16s rRNA	0.19	0.13	1.00	1.00	0.19	0.76	Dunn

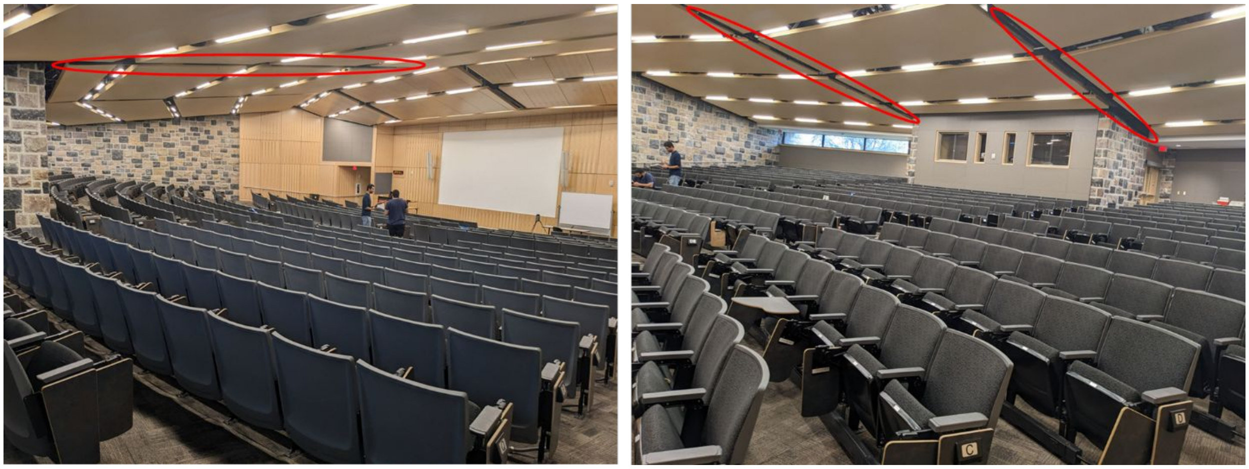

Figure S1. Photographs of the lecture hall from the a) back of the room and b) front of the room. Ceiling vents/diffusers that are visible are circled in red.

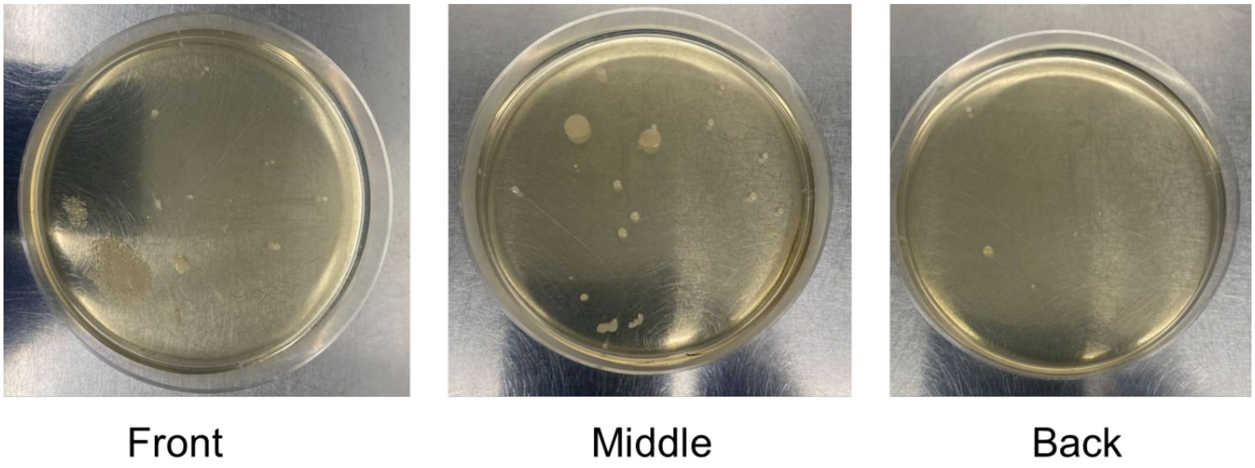

Figure S2. Example bacterial growth plates from the front, middle, and back of the lecture hall.

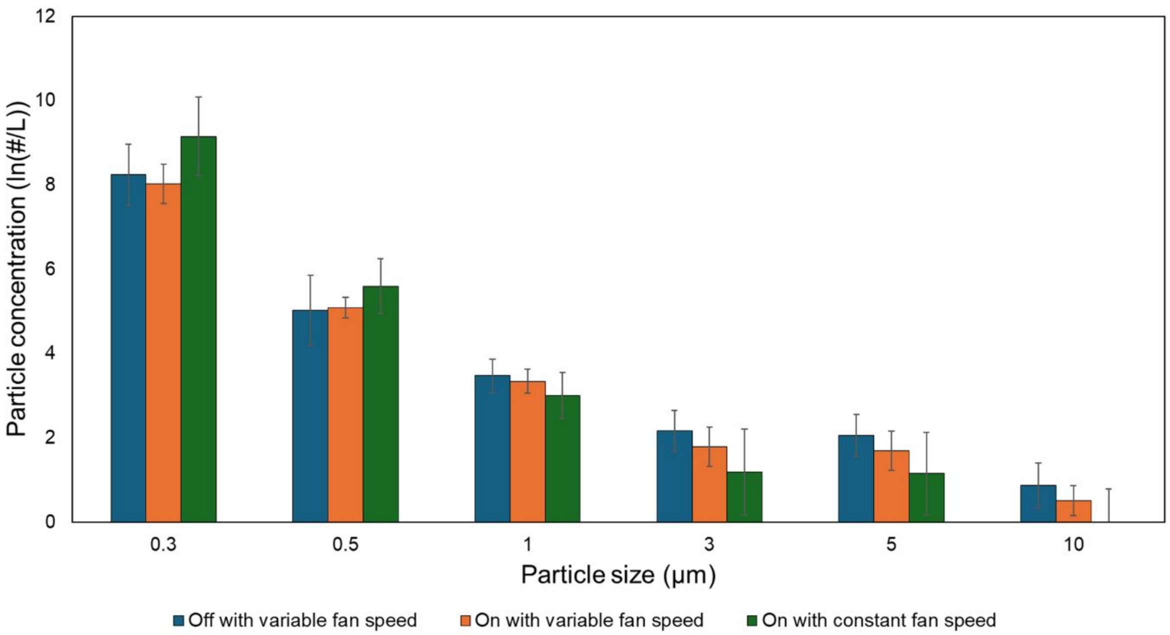

Figure S3. Particle number concentrations by size, log transformed. The size shown is the lower bound of the range measured.

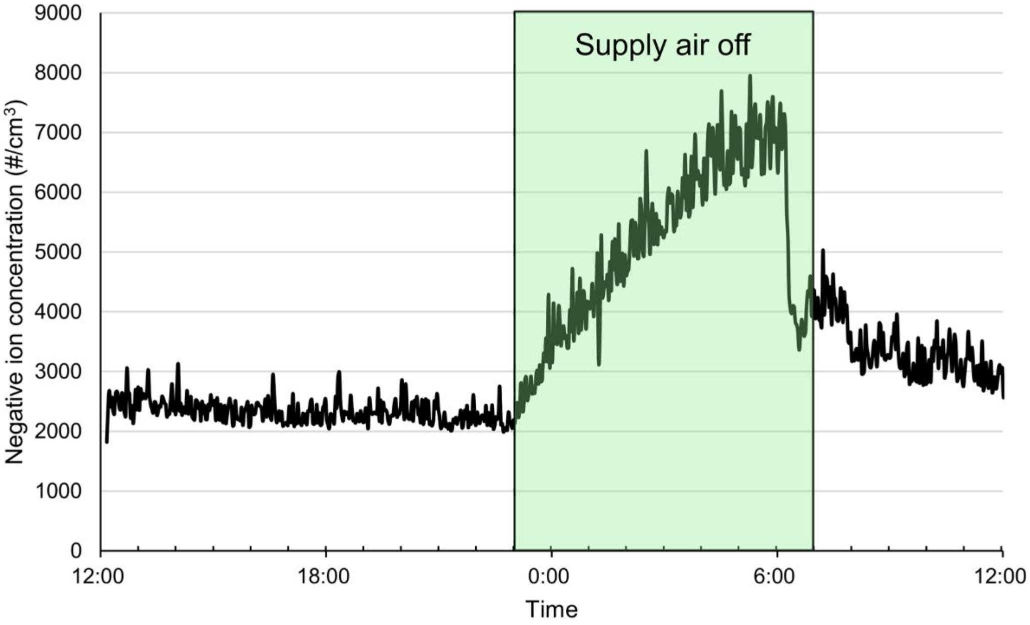

Figure S4. Negative ion concentrations increased at nighttime when the supply air was off.

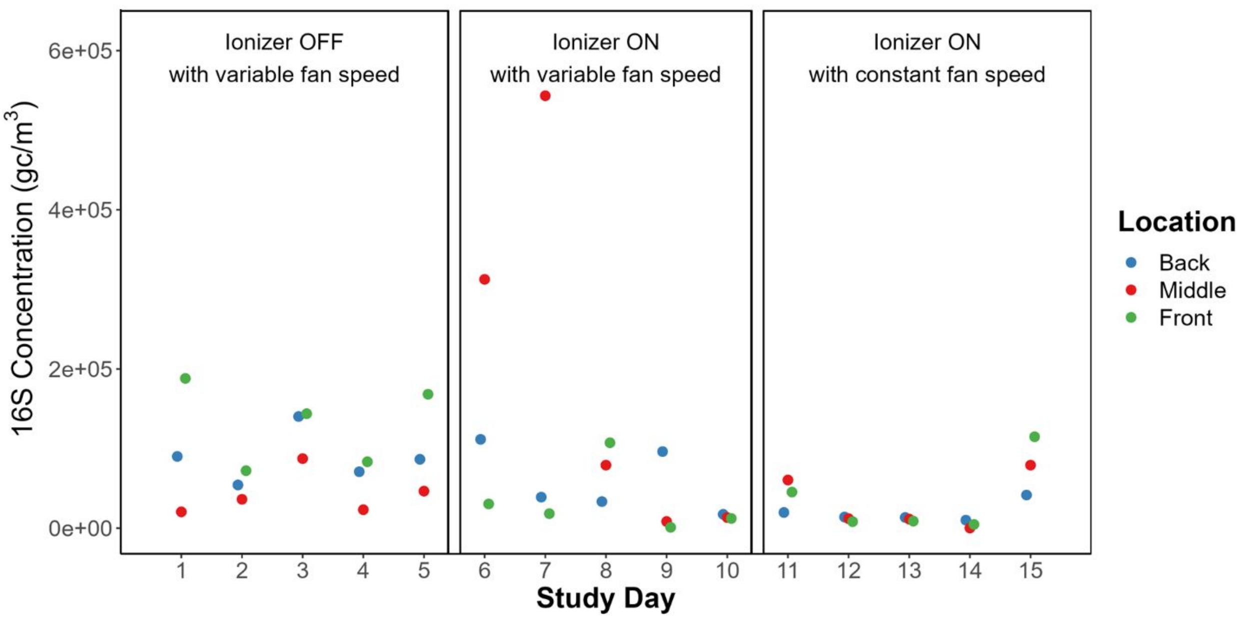

Figure S5. 16S rRNA gene copy concentrations plotted on a linear scale.

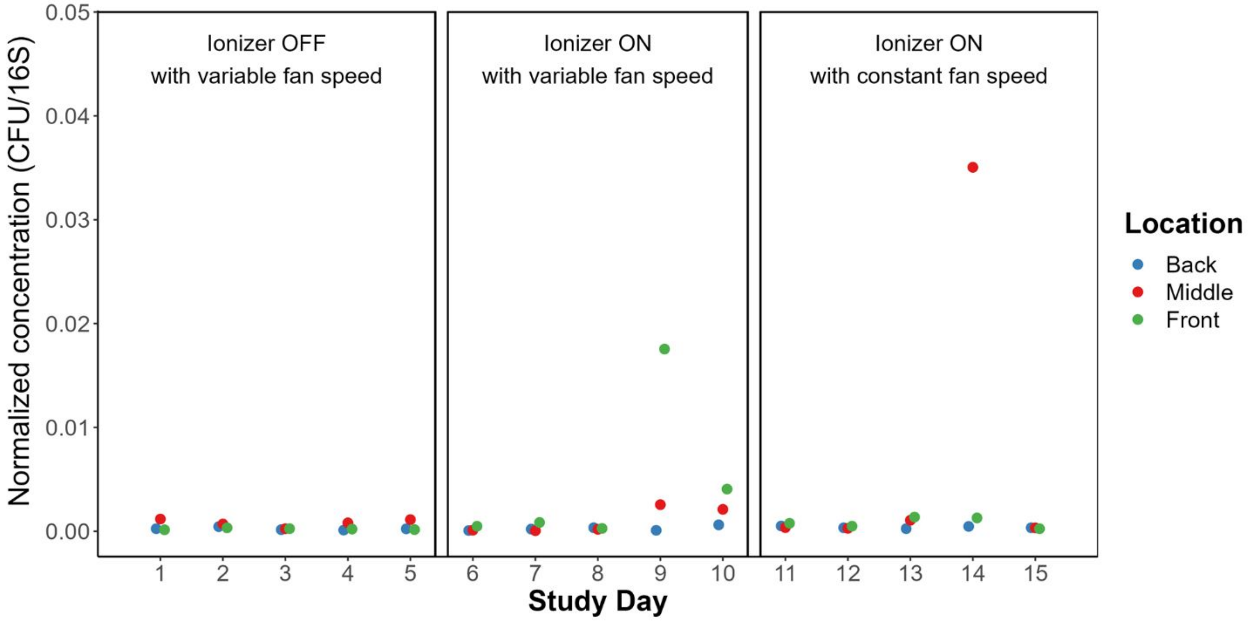

Figure S6. Normalized bacteria concentrations (CFU per 16S rRNA gene copy) plotted on a linear scale.

(a)

	In-duct ions	Fan speed	In-duct CO_2	In-duct RH
In-room ions	0.00	0.00	0.00	0.25
In-duct ions		0.00	0.00	0.00
Fan speed			0.00	0.00
In-duct CO_2				0.00

(b)

	Fan speed	Fan speed ▲	In-duct CO2	In-duct RH	Particle concentration	In-room ions (-)	In-room ions (+)	Occupancy	Total bacteria	Culturable bacteria
In-duct ions	0.16	0.04	0.27	0.07	0.26	0.60	0.01	0.97	0.05	0.14
Fan speed		0.00	0.14	0.77	0.10	0.95	0.19	0.28	0.17	0.38
Fan speed ▲			0.05	0.35	0.02	0.54	0.28	0.28	0.07	0.09
In-duct CO2				0.47	0.00	0.06	0.96	0.01	0.24	0.00
In-duct RH					0.98	0.12	0.58	0.92	0.85	0.38
Particle concentration						0.08	0.77	0.02	0.01	0.01
In-room ions (-)							0.06	0.01	0.07	0.01
In-room ions (+)								0.22	0.87	0.96
Occupancy									0.04	0.01
Total bacteria										0.08

Figure S7. p-values for correlation plots for variables measured (a) continuously and (b) only during the 1-hour sampling period. Total bacteria are quantified in terms of 16S rRNA gene copies. Triangle (▲) denotes measurements from the hour before sampling. In-duct ion measurements may represent positive or negative ions or both.

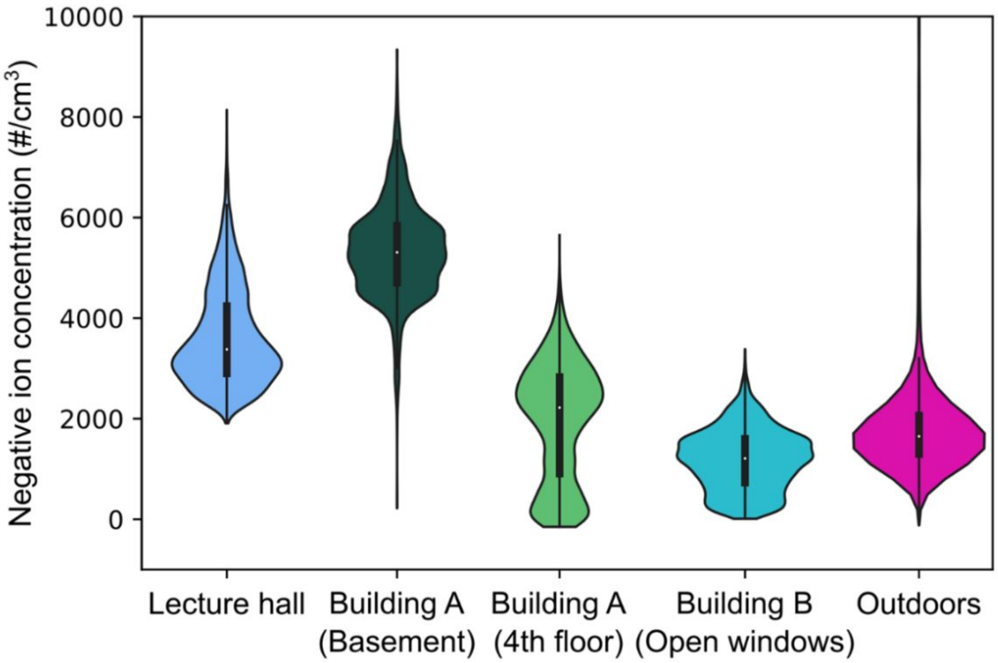

Figure S8. Negative ion concentrations in other rooms and buildings, including a basement, a fourth-floor classroom, a ground-level room with open windows, and an outdoor setting.
